# Surveillance of catheter-associated bloodstream infections: development and validation of a fully automated algorithm

**DOI:** 10.1186/s13756-024-01395-4

**Published:** 2024-04-10

**Authors:** Gaud Catho, Loïc Fortchantre, Daniel Teixeira, Murielle Galas-Haddad, Filippo Boroli, Marie-Noëlle Chraïti, Mohamed Abbas, Stephan Harbarth, Niccolò Buetti, Swissnoso group, Carlo Balmelli, Delphine Berthod, Philipp Jent, Jonas Marschall, Hugo Sax, Matthias Schlegel, Alexander Schweiger, Laurence Senn, Rami Sommerstein, Sarah Tschudin-Sutter, Nicolas Troillet, Danielle Vuichard-Gysin, Andreas F. Widmer, Aline Wolfensberger, Walter Zingg

**Affiliations:** 1 Infection Control Programme and World Health Organization Collaborating Centre, Geneva University Hospitals and Faculty of Medicine, Geneva, Switzerland; 2 Infectious Diseases Division, Central Institute, Valais Hospital, Sion, Switzerland; 3 Intensive Care Unit Division, Geneva University Hospitals and Faculty of Medicine, Geneva, Switzerland; 4 MRC Centre for Global Infectious Disease Analysis, Jameel Institute, School of Public Health, Imperial College London, London, UK; 5 INSERM, IAME, Université Paris-Cité, Paris, 75006 France

**Keywords:** Sensitivity, Specificity, Healthcare associated infections, CLABSI, Bloodstream infection, Catheter-infection, Intensive care unit, Digital, Internal validation

## Abstract

**Background:**

Most surveillance systems for catheter-related bloodstream infections (CRBSI) and central line-associated bloodstream infections (CLABSI) are based on manual chart review. Our objective was to validate a fully automated algorithm for CRBSI and CLABSI surveillance in intensive care units (ICU).

**Methods:**

We developed a fully automated algorithm to detect CRBSI, CLABSI and ICU-onset bloodstream infections (ICU-BSI) in patients admitted to the ICU of a tertiary care hospital in Switzerland. The parameters included in the algorithm were based on a recently performed systematic review. Structured data on demographics, administrative data, central vascular catheter and microbiological results (blood cultures and other clinical cultures) obtained from the hospital’s data warehouse were processed by the algorithm. Validation for CRBSI was performed by comparing results with prospective manual BSI surveillance data over a 6-year period. CLABSI were retrospectively assessed over a 2-year period.

**Results:**

From January 2016 to December 2021, 854 positive blood cultures were identified in 346 ICU patients. The median age was 61.7 years [IQR 50–70]; 205 (24%) positive samples were collected from female patients. The algorithm detected 5 CRBSI, 109 CLABSI and 280 ICU-BSI. The overall CRBSI and CLABSI incidence rates determined by automated surveillance for the period 2016 to 2021 were 0.18/1000 catheter-days (95% CI 0.06–0.41) and 3.86/1000 catheter days (95% CI: 3.17–4.65). The sensitivity, specificity, positive predictive and negative predictive values of the algorithm for CRBSI, were 83% (95% CI 43.7–96.9), 100% (95% CI 99.5–100), 100% (95% CI 56.5–100), and 99.9% (95% CI 99.2–100), respectively. One CRBSI was misclassified as an ICU-BSI by the algorithm because the same bacterium was identified in the blood culture and in a lower respiratory tract specimen. Manual review of CLABSI from January 2020 to December 2021 (*n* = 51) did not identify any errors in the algorithm.

**Conclusions:**

A fully automated algorithm for CRBSI and CLABSI detection in critically-ill patients using only structured data provided valid results. The next step will be to assess the feasibility and external validity of implementing it in several hospitals with different electronic health record systems.

**Supplementary Information:**

The online version contains supplementary material available at 10.1186/s13756-024-01395-4.

## Background

Based on data collected by the European Centre for Disease Prevention and Control (ECDC), 4.5 million healthcare-associated infections (HAIs) have been estimated to occur each year in European hospitals [1, 2], with a large proportion of these infections being preventable [3]. Hospital-acquired bloodstream infections (BSIs) and hospital-acquired pneumonia accounted for 60% of the total burden of HAIs [1]. Intravascular catheters are widely used in intensive care unit (ICU) patients, with 70% having a central vascular catheter (CVC) on any given day [4], making them particularly vulnerable to central line-associated bloodstream infection (CLABSI). Intravascular catheter infections are associated with increased mortality, morbidity, hospital length of stay and costs [5].

Surveillance of HAIs, and particularly CLABSI, is necessary to achieve optimal prevention and has been identified as one of the most cost-effective prevention measures in itself [6]. Most HAI surveillance systems rely on manual review of medical charts by trained healthcare professionals. Such surveillance systems are time-consuming, prone to errors and have limited interrater reliability [7]. With the widespread adoption of electronic health records (EHRs), electronic data are becoming routinely available and can thus be used for automated surveillance purposes. However, automated surveillance systems for CLABSI, has been mostly limited to research settings or to single institutions [8]. In Switzerland, the surveillance of CLABSI is limited to local initiatives of hospitals and is performed manually [9]. The Swiss Federal Office of Public Health has mandated the Swiss National Center for Infection Control to develop and implement a national surveillance system to monitor the incidence of CLABSI in acute care hospitals. As a first step, a fully automated surveillance algorithm was developed to monitor catheter-related BSI (CRBSI) and CLABSI incidence rates in ICUs.

The main objective of the current study was to assess the validity of a fully automated algorithm for CRBSI and CLABSI surveillance in the ICU of the largest tertiary care centre in Switzerland.

## Methods

### Setting, patients and catheters

This study was conducted in the adult ICU of Geneva University Hospitals, a tertiary care hospital located in Geneva, Switzerland, with 10 sites, 2008 beds, and approximately 60 000 admissions per year. The adult ICU is a mixed medical-surgical ICU with a total of 32 beds and provides care for approximately 2500 patients annually with an average length of stay of 4 days. All adult patients (> 18 years old) with at least one stay in the ICU from January 1, 2016, to December 31, 2021, were included. All short-term CVCs in situ while the patient was in the ICU were included [10]. All long-term CVCs (e.g., Broviac®), peripherally inserted central catheters, dialysis catheters and arterial catheters were excluded. For validation of the algorithm, we used prospectively collected data from the routine surveillance program in place at the Geneva University Hospitals [11]. For more than 25 years, the Infection Prevention and Control (IPC) team has been conducting hospital-wide prospective surveillance of all healthcare-associated BSIs. For each healthcare-associated BSI episode, data on the source of infection and clinical and microbiological characteristics are routinely collected manually by the IPC team. The IPC team members are alerted to every new positive blood culture result by the central microbiology laboratory, and they prospectively follow-up and investigate the sources of healthcare-associated episodes. All episodes occurring more than 48 h after hospital admission or within 10 days of a previous hospitalization are investigated.

### Data sources for the automated surveillance and the manual surveillance

For the automated surveillance, patient-level data (age, sex, admission and discharge dates, mortality at day 30), individual-level CVC data (date of insertion and removal, ward of insertion, insertion site, and dwell-time) and microbiological data (blood culture results, other culture results, specimen collection dates) were extracted from the EHR. For the comparator (manual BSI surveillance), all BSI data were extracted from the hospital. 

### Definitions

BSI were classified according to adapted ECDC definition criteria, as those used in the European point prevalence study [12–14]. The primary outcome, CRBSI, was defined as a BSI that occurred at any time point from the day of catheter insertion up to 48 h after catheter removal, and a blood culture result with the same microorganism as a quantitative CVC tip culture of 10^3^ colony-forming units (CFU) per mL or greater [15] (or semiquantitative central venous catheter culture > 15 CFU) [12]. Of note, the following criteria were not included in the automated algorithm for CRBSI: *(a)* quantitative blood culture ratio CVC blood sample/peripheral blood sample > 5; *(b)* differential time of blood culture positivity (DTP); *(c)* positive culture with the same microorganism from pus at the insertion site and *(d)* clinical criterion of improvement within 48 h of catheter removal. Criteria (a) and (b) were not implemented in the automated algorithm because they were not systematically provided by our microbiology laboratory. Only CRBSI episodes that started 48 h after ICU admission were considered (the initial positive blood culture of the episode was collected after the patient has spent a minimum of 48 h in the ICU).

Secondary outcomes included CLABSI and ICU-onset BSI. CLABSI was defined as a BSI that occurred from day of catheter insertion until 48 h after catheter removal, with the absence of positive culture from other specimens with the same microorganism within an interval of 72 h before/after the first positive blood culture of the episode. This rule was not applied if the microorganism was a common commensal, even though the rule to classify a common commensal as a true pathogen was fulfilled. The types of other specimens considered were restricted to urine, respiratory tract, bone and joint, abdominal, and central nervous system specimens (full list provided in Appendix, Suppl Table 1) based on previous work performed by our group [16]. Only BSI episodes that started 48 h after ICU admission were also considered. Finally, ICU-onset BSI was defined as a BSI episode for which the first positive blood culture of the episode was collected after the patient has spent a minimum of 48 h in the ICU, regardless of the presence of a CVC.

A common commensal was considered as a true pathogen when the same common commensal was present in at least 2 positive blood cultures within 48 h. Common commensal included, among other, coagulase-negative staphylococci, *Bacillus* species, *Propionibacterium* species, *Corynebacterium* species, or *Micrococcus* species (as defined by the CDC NHSN [“NHSN Organism Category”] [17]).

A BSI episode was defined as any positive blood culture with the same pathogen within a time-window of 14 days (counted in hours). To limit complexity, polymicrobial blood cultures were considered as separate episodes. Negative blood cultures were not considered to define an episode. A set of blood cultures (two vials) was counted as one blood culture. Catheter days were counted in hours. CVC days of two or more concurrent CVCs were all counted. Catheter insertion and removal dates were replaced with ICU admission and discharge dates, respectively, when missing.

### Development of the fully automated algorithm

**Fig. 1:**
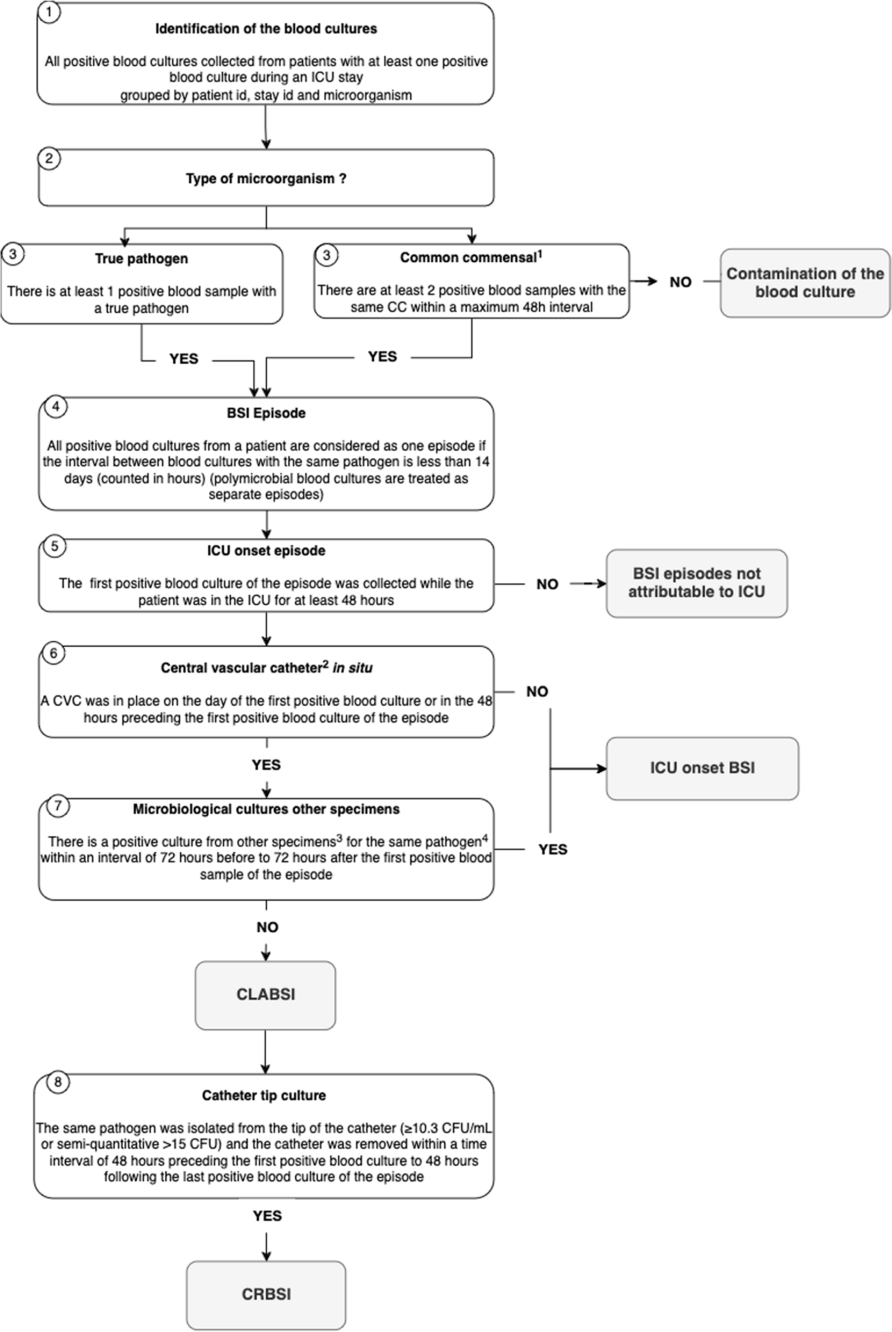
Fully automated algorithm developed for CRBSI, CLABSI and intensive care unit (ICU) onset BSI detection for patients in the ICU. 1. List of common commensals from the CDC NHSN. 2. Only short term central vascular catheters are considered. 3. Specimens included : respiratory samples, urinary samples, central nervous system samples, abdominal samples, bone and joints samples. 4. This rule applies only to true pathogens and not to common commensal (even if classified as true pathogen on step 3). ICU: intensive care unit; BSI: bloodstream infection; CVC: central vascular catheter; CLABSI: central line associated Bloodstream Infection; CRBSI: Catheter related bloodstream infection; CC: common commensals

The IPC team in collaboration with an information technology (IT) team developed a fully automated algorithm for CLABSI/CRBSI detection (Fig. 1), which was based on a systematic review, meta-analysis and meta-regression that identified relevant parameters to be implemented [16]. The algorithm was built through an iterative process with a regular manual check using clinical use cases for each parameter of the algorithm.

The primary outcome identified by the algorithm was CRBSI, as described above. CRBSI has been identified as the gold-standard patient-oriented endpoint, as it has the best construct validity to establish causality between a BSI and the catheter and because it is significantly associated with increased mortality [18]. The two secondary outcomes identified by the algorithm were CLABSI and ICU-onset BSI.

### Validation and statistical methods

Incidence was calculated using catheter-days as a denominator for CRBSI and CLABSI and patient-days for ICU-BSI. For CRBSI, we calculated estimates of sensitivity, specificity, negative and positive predictive values of the automated algorithm detection compared to the reference standard (manual surveillance using ECDC definitions), from 1 January 2016 to 31 December 2021. Root cause analysis was performed for all discrepancies between the manual and automated monitoring results.

For validation of the secondary outcome (*i.e.*, CLABSI), each episode identified by the algorithm from 1 January 2020 to 31 December 2021 was manually reviewed by an IPC pharmacist (MGH) and/or an ID specialist (GC). Each episode was classified as true positive or false positive, according to the aforementioned definitions. For validation of the ICU-BSI, a random sample of the episodes (10% of the episodes) identified by the algorithm from 1 January 2019 to 31 December 2021 was manually reviewed following the same method.

The calculation of sensitivity, specificity, negative and positive predictive values of the fully automated surveillance system was performed according to standard epidemiological methods [19]. Confidence intervals for the specificity, sensitivity, positive and negative predictive values were performed using the Wilson score interval method. All calculations were performed with R (R foundation, version 4.1.3).

### Ethics

This surveillance was conducted as part of the routine quality improvement activities of our infection control program. Thus, institutional review board approval from the Commission Cantonale d’Éthique de la Rercherche de Genève, as well as individual consent, was not required, according to the definition of research in the Swiss Human Research Act. All data were anonymized.

## Results

From 1 January 2016 to 31 December 2021, a total of 853 positive blood cultures from 346 ICU patients were identified. The median age was 61.7 years (IQR: 50–70) and 205 (24%) of the 853 positive blood cultures were collected from female patients. The algorithm identified 5 CRBSI episodes, 109 CLABSI episodes and 280 ICU-onset BSI episodes among 5, 96 and 223 patients, respectively (Suppl Figure 1). The numbers of catheter-days and patient-days for the same period were 28’267 and 50’929, respectively. The demographics for each type of episode are presented in Table 1. The most frequent pathogens isolated are presented in appendix (Suppl Figure 2).

**Table 1:** Demographics of the study population presented by episodes

Number of episodes	CRBSI (*N* = 5)	CLABSI (*N* = 109)	ICU-BSI (*N* = 280)	Positive blood cultures from patients with an ICU stay (*N* = 853)
**Number of patients**	5	96	223	346
**Age median [IQR]**	58.3 [32.3–68.5]	63.8 [50.6–70.9]	63.6 [50.5–71.7]	61.7 [50–70]
**Female N (%)**	2 (40%)	28 (26.4)	73 (26.8%)	205 (24%)
**30-day mortality N (%)**	0	39 (35.4)	84 (30)	200 (23.4)

Data are presented by episode: one patient can have several episodes (e.g. 280 ICU-BSI episodes occurred in 223 patients)

*IQR* Interquartile range, *N* Number, *ICU* Intensive care unit, *CRBSI* Catheter related bloodstream infection, *CLABSI* Central line associated bloodstream infection, *BSI* Bloodstream infection, *%* Percent

### Incidence of CRBSI, CLABSI and ICU-onset BSI

The overall incidence rate of CRBSI from the period 2016 to 2021 was 0.18/1000 catheter-days (95% CI 0.06–0.41). The overall incidence rate of CLABSI from 2016 to 2021 was 3.86/1000 catheter-days (95% CI: 3.17–4.65). The overall incidence rate of ICU-onset BSI from 2016 to 2021 was 5.50/1000 patient-days (95% CI: 4.87–6.18) (Table 2).

**Table 2:** Incidence rate of CRBSI/1000 catheter days, CLABSI/1000 catheter days and ICU-BSI/1000 patient days

Year	Patient days	Catheter days	CRBSI	CLABSI	ICU onset BSI	ICU-onset BSI/1000 patient-days (95% CI)	CRBSI/1000 catheter-days (95%CI)	CLABSI/1000 catheters-days (95%CI)
**2016**	8839	3946	0	20	44	4.98 (3.62–6.68)	0	5.07 (3.10–7.83)
**2017**	8998	4253	2	17	46	5.11 (3.74–6.82)	0.47 (0.06–1.66)	4.00 (2.33–6.40)
**2018**	7206	3150	0	12	42	5.69 (4.08–7.72)	-	3.81 (1.97–6.65)
**2019**	7641	4146	0	9	34	4.45 (3.08–6.22)	-	2.17 (0.99–4.12)
**2020**	8597	5624	0	16	52	6.16 (4.62–8.06)	-	2.84 (1.63–4.62)
**2021**	9648	7148	3	35	62	6.43 (4.93–8.24)	0.42 (0.09–1.23)	4.90 (3.41–6.81)
**Total**	50,929	28,267	5	109	280	5.50 (4.87–6.18)	0.18 (0.06–0.41)	3.86 (3.17–4.65)

*N* Number, *ICU* Intensive care unit, *CRBSI* Catheter related bloodstream infection, *CLABSI* Central line associated bloodstream infection, *BSI* Bloodstream infection, *%* Percent, *CI* Confidence interval

### Validation of CRBSI

From 1 January 2016 to 31 December 2021, the algorithm identified 5 CRBSIs in patients in the ICU for at least 48 h. Manual surveillance identified 6 CRBSI. Five CRBSI were correctly identified by the algorithm. Root-cause analysis showed that the algorithm did not identify 1 patient as having a CRBSI because the patient had a positive respiratory specimen with the same bacteria (*Pseudomonas aeruginosa*) as in the blood culture in the defined time window. For CRBSI detection, the sensitivity of the algorithm was 83.3% (95% CI 43.7–96.9) and the specificity was 100% (95% CI 99.5–100). The positive and negative predictive values were 100% (95%CI: 56.5–100) and 99.9% (95%CI: 99.2–100), respectively (Table 3).

**Table 3:** Validation of the fully automated algorithm for CRBSI

***Cross tabulation of manual Surveillance and fully automated surveillance ****(validation sample (n = 853))*		Manual surveillance	
		CRBSI	No CRBSI
**Fully automated surveillance**	CRBSI	5 (TP)	0 (FP)
	No CRBSI	1 (FN)	847 (TN)
***Performance of the fully automated algorithm***			
Sensitivity	83.3% (95%CI: 43.7–96.9)		
Specificity	100% (95%CI: 99.5–100)		
Positive predictive value	100% (95%CI: 56.5–100)		
Negative predictive value	99.9% (95%CI: 99.2–100)		

*CI* Confidence interval, *CRBSI* Catheter related bloodstream infection, *CLABSI* Central line associated bloodstream infection, *TP* True positive, *FP* False positive, *FN* False negative, *TN* True negative

### Validation of CLABSI

From 1 January 2020 to 31 December 2021, the algorithm identified 51 CLABSIs in patients in the ICU for at least 48 h. Each parameter of the algorithm was checked manually for the 51 CLABSI and was correct.

### Validation of ICU-BSI

From 1 January 2020 to 31 December 2021, the algorithm identified 280 ICU-BSI in patients in the ICU for at least 48 h. Among them, 28 were randomly selected. Each algorithm step checked manually for these 28 ICU-BSI was correct.

## Discussion

Our study demonstrates the internal validity of a fully automated algorithm for CRBSI detection in the ICU population of a large tertiary care hospital. Compared to manually performed surveillance, the fully automated surveillance system, based only on routine clinical and administrative data extracted from the EHR, provided excellent specificity and very good sensitivity.

Despite advances in EHR implementation, automated surveillance for HAI is still in its infancy, as demonstrated by a recent European survey conducted by the PRAISE (Providing a Roadmap for Automated Infection Surveillance in Europe) network [20]. Very few studies have reported the validation of a fully automated CLABSI detection algorithm. A recent systematic review identified only five studies reporting automated surveillance of CLABSI/CRBSI [16], including data from 2004 to 2015. Moreover, while all studies identified only one outcome (CLABSI for 4 studies and CRBSI for one study), we have developed an algorithm that identified 3 different indicators (CRBSI, CLABSI and ICU-BSI). In Table 4, we provide a set of key data of our algorithm, including lessons learned during the development process, as suggested by the PRAISE network [20].

**Table 4:** Main features of the fully automated algorithm for CRBSI/CLABSI detection according to PRAISE

**Type of system**	Fully automated
**HAI targeted**	Intravascular catheter infections (definitions adapted from ECDC)
**Date sources**	Electronic Health Record
**Validation method**	Comparison with prospective manual surveillance of BSI conducting according to ECDC definitions
**Comparator**	Manual surveillance of bloodstream infections over a 6-year period (2016 to 2021)
**Data type included**	Administrative data, microbiology lab data, individual intravascular catheters data extracted from data EHR
**Patient population**	All adult patients admitted to the intensive care unit
**Indicators**	CRBSI, CLABSI, ICU-onset BSI
**Denominators**	Catheter-days and patient-days
**Sensitivity**	83% (95%CI: 43.7–96.9)
**Specificity**	100% (95%CI: 99.5–100)
**Lessons learned**	- Several cut-offs for different parameters in the algorithm were set arbitrarily and would require further in-depth sensitivity analyses (e.g., delay between two blood cultures with the same common contaminant to consider the episode; delay to consider positive specimens with the same bacteria as in the blood cultures to exclude a CRBSI). - Some ECDC rules were not transposable in a fully automated algorithm because of the lack of availability or accuracy of the data in the IT system: (e.g., quantitative blood culture ratio CVC blood sample/peripheral blood sample; differential time of blood culture positivity) - Some data are difficult to capture in a fully automated algorithm because of the lack of standardisation (e.g., culture from pus from the insertion site of the catheters are frequently mislabelled and difficult to identify in the microbiology database).

*BSI* Bloodstream infections, *CVC* Central vascular catheter, *ECDC* European Center Diseases Control, *CRBSI* Catheter related bloodstream infection, *CLABSI* Central line associated bloodstream infection, *ICU* Intensive care unit

Intravascular catheter infections are suitable for *fully automated* surveillance because of well-established definitions, largely based on data that can be captured in a structured way by the EHR, and because CLABSI relies primarily on positive blood cultures, which provide a strong and easily identifiable criterion. The main advantages of automated surveillance at hospital level include time efficiency/workload reduction, which could lead to reallocation of saved IPC resources, the inclusion of large amounts of data to provide a more comprehensive overview, and the ability to perform real-time surveillance and therefore targeted infection prevention interventions [7, 21]. Likely, bedside staff would be more involved in HAI prevention following effective and almost live feedbacks on patients’ outcomes [22–24].

Our algorithm involved a limited number of rules and data inputs, was based on structured data only, and, by definition, did not include manual assessment. In this context, fully automated surveillance may have a greater potential for standardization and is more likely to be used outside a single institution. With the overarching aim of implementing a nationwide surveillance in Swiss acute care hospitals and enabling benchmarking between healthcare facilities, we designed a fully automated algorithm with limited complexity to facilitate wider implementation, local validation and long-term maintenance. The need for IT is much lower compared to the use of unstructured data, which would require natural language processing. The implementation of natural language processing-based surveillance strategies is currently limited by, among other things, the complexity of unstructured data and variable documentation practices [25, 26]. Nevertheless, for a wider implementation of our tool, it will be necessary to achieve high level of data standardization between hospitals, which remains a challenge. Hospitals in Switzerland, as in many countries worldwide, use a wide variety of commercial or in-house EHRs, and most structured data are not coded in a common referential such as SNOMED-CT [9], although recent progress has been made through the SPHN network [27].

The reported incidence rate of CLABSI in the U.S by the CDC NSHN surveillance network was 1.05 / 1,000 catheter days in 2022, and the incidence rate of CLABSI reported by the ECDC for ICU was 3.4 /1,000 catheter days in 2019 [28]. If we compare these results with those from our automated surveillance, we can observe a higher incidence for CLABSI and a lower for CRBSI. CRBSI requires a positive catheter tip culture and therefore a lower incidence is expected compared to the CLABSI rate from the CDC NSHN surveillance. Our automated CLABSI identification relies on the absence of positive cultures from other specimens (which would attribute the BSI to another source of infection). We have voluntarily restricted the type of specimens included in the algorithm to avoid attributing a BSI to a false infection. Including any type of positive specimen (e.g. ‘superficial swab’) would have resulted in a lower CLABSI rate. In addition, we cannot exclude the possibility that secondary infections, such as surgical site infections, may not always have a positive culture result from the primary site of infection, which would also lead to an overestimation of the CLABSI rate. Comparisons of incidence rates from the automated surveillance with manual surveillance should be interpreted with caution because of differences in definition. The overarching aim of our surveillance was to establish a national standard using automated systems for benchmarking purposes, but not for benchmarking with manually collected surveillance data.

Our study has several limitations. First, our algorithm identifies polymicrobial blood cultures as separate BSI episodes. This choice was made to limit the complexity of the algorithm. However, this rule may overestimate CRBSI and CLABSI incidence rates, as a single episode could be counted several times. Second, some ECDC criteria such as quantitative blood cultures ratio, DTP and positive culture of pus from catheter insertion site were not included, mainly due to the IT challenges and lack of reliability of these data, which may conversely underestimate the true incidence of CRBSI. Nevertheless, paired quantitative blood cultures are a limited diagnostic method by the lack of standardized cut-off points and are rarely performed in most laboratories. Moreover, the inclusion of DTP has been shown to have a limited impact on the detection of CRBSI in short-term CVCs [18], with poor sensitivity and limited specificity [29]. Third, our algorithm misclassified one event because the same pathogen was identified in the blood culture and in a lower respiratory tract specimen within the predefined interval. Manual review of the patient chart did not identify a lower respiratory tract infection and classified the positive respiratory specimen as a colonization (false negative). This example illustrates the lower specificity of a fully automated algorithm compared to a semi-automated system. Compared with a semi-automated surveillance system, this could result in a possible reduction in clinical relevance and clinician buy-in [7]. Fourth, although based on a systematic review and a meta-regression analysis to identify the best performing parameters, several cut-off parameters (such as the time window to consider two common commensals as a true pathogen) were arbitrary or based on existing definitions from CDC/NSN and ECDC. Fifth, a fully automated system designed to limit complexity may lose clinical relevance and limit its use in measuring the appropriateness and effectiveness of preventive interventions. Finally, we limited the scope of our study to critically-ill patients in a high-resource setting, thus limiting the generalizability of our results to that population. However, this population is at particular risk of intravascular catheter infections due to frequent exposure to CVCs [4]. Still, this surveillance approach could easily be applied to other populations such as acute care patients.

## Conclusions

Our study provides solid evidence of the good performance of a fully automated algorithm for the detection of CRBSI and CLABSI in ICU patients. The next step will be to perform external validation of the automated algorithm by implementing it in other hospitals with different EHR systems.

### Supplementary Information

**Additional file 1: Suppl Figure 1. **Proportion of the 5 most frequent pathogens by type of episode (CRBSI, CLABSI, ICU-BSI and All BSI). **Suppl Table 1.** List of specimens considered in the CLABSI and CRBSI definition.

## Data Availability

The datasets used and/or analyzed during the current study are available from the corresponding author on reasonable request.
