# Supplementary material for: Surveillance of catheter-associated bloodstream infections: development and validation of a fully automated algorithm
Source: Antimicrob Resist Infect Control. 2024 Apr 10;13:38. doi: 10.1186/s13756-024-01395-4 (PMC11007875; doi:10.1186/s13756-024-01395-4)
Supplement: Supplementary file 1 — Additional file 1: Suppl Figure 1. Proportion of the 5 most frequent pathogens by type of episode (CRBSI, CLABSI, ICU-BSI and All BSI). Suppl Table 1. List of specimens considered in the CLABSI and CRBSI definition. [file 13756_2024_1395_MOESM1_ESM.zip › Appendix.docx]

**Appendix**

**Surveillance of catheter-associated bloodstream infections: development and validation of a fully automated algorithm**

**Suppl Figure 1.** Proportion of the 5 most frequent pathogens by type of episode (All BSI, CRBSI, CLABSI and ICU-BSI)

**
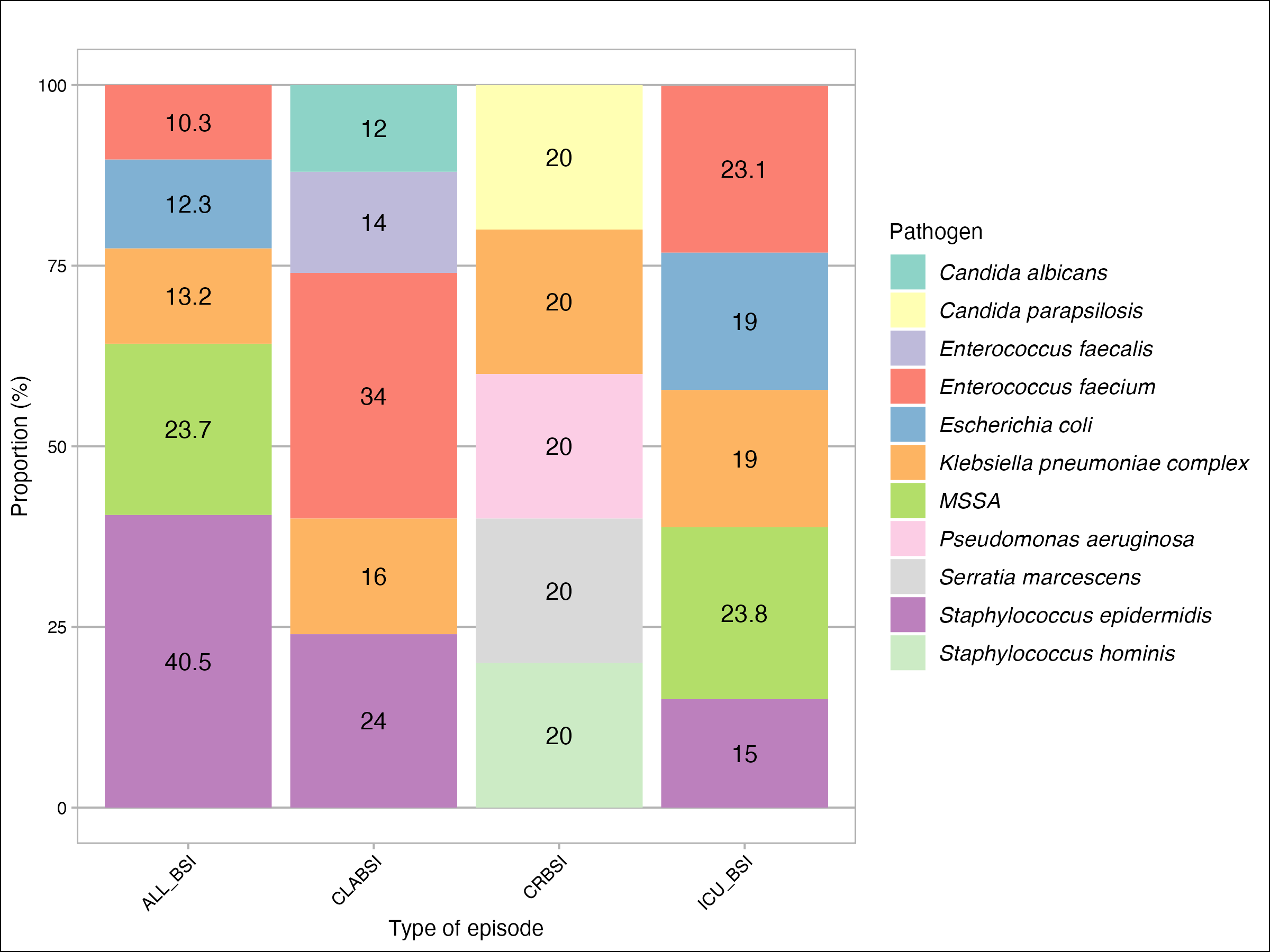
**

MSSA : methicillin susceptible *Staphylococcus aureus;* MRSA: methicillin resistant *Staphylococcus aureus*

**Supp. Figure 2.**

**Suppl Table 1.** List of specimens considered in the CLABSI definition.

| **Specimen categories** | **Specimens** |
| --- | --- |
| Respiratory tract | Bronchoalveolar lavage, tracheal aspiration, bronchial aspiration, sputum, pleural fluid, lung biopsy |
| Urinary tract | Urine |
| Abdominal | Peritoneal fluid, intra-abdominal abscess, biliary fluid |
| Central Nervous System | Cerebrospinal fluid |
| Bone and joint | Bone biopsy, joint fluid, synovial fluid, orthopedic material |
