# Supplementary figures and images for: Surveillance of catheter-associated bloodstream infections: development and validation of a fully automated algorithm
Source: Antimicrob Resist Infect Control. 2024 Apr 10;13:38. doi: 10.1186/s13756-024-01395-4 (PMC11007875; doi:10.1186/s13756-024-01395-4)

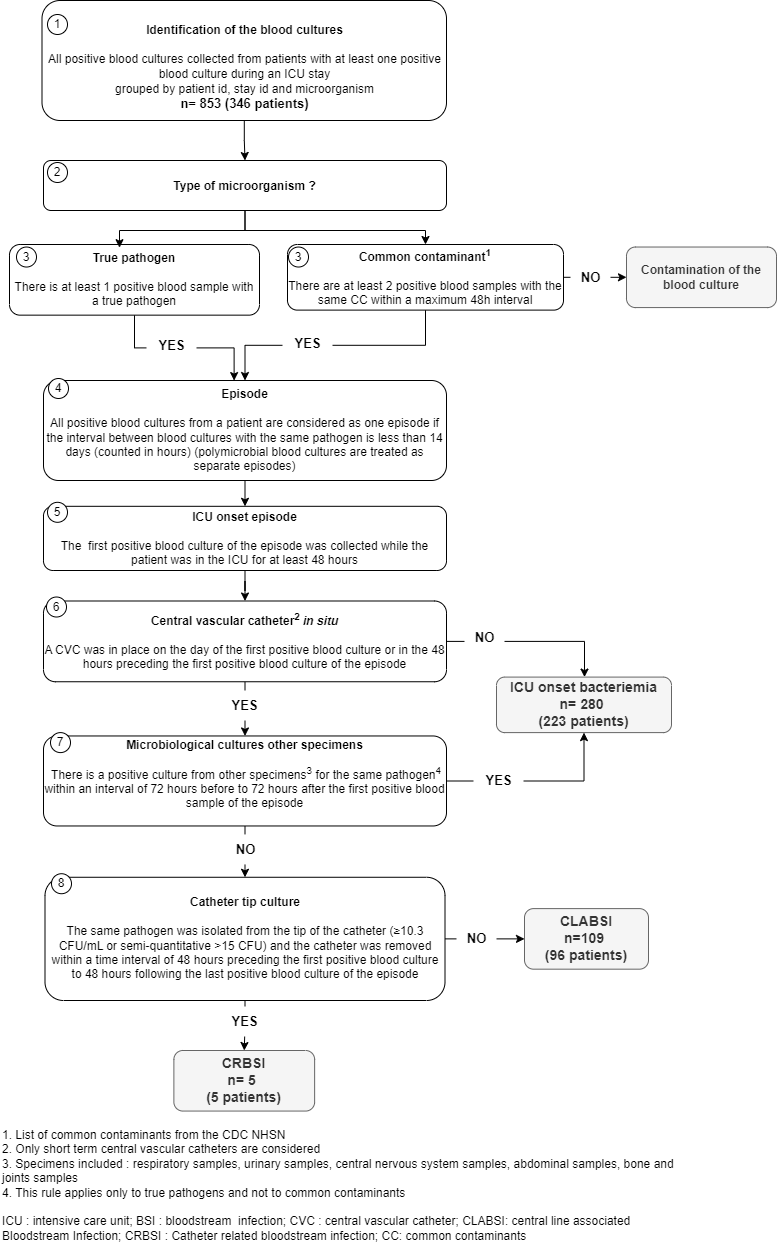

Supplement: Supplementary file 1 — Additional file 1: Suppl Figure 1. Proportion of the 5 most frequent pathogens by type of episode (CRBSI, CLABSI, ICU-BSI and All BSI). Suppl Table 1. List of specimens considered in the CLABSI and CRBSI definition. [file 13756_2024_1395_MOESM1_ESM.zip › Suppl.figure.1.png]
